# BV score differentiates viral from bacterial-viral co-infection in adenovirus PCR positive children

**DOI:** 10.3389/fped.2022.990750

**Published:** 2022-11-01

**Authors:** Michal Stein, Ma’anit Shapira, Ellen Bamberger, Irena Chistyakov, Daniel Dumov, Isaac Srugo, Michal Stein, Louis J. Bont, Adi Klein

**Affiliations:** ^1^ Pediatric Infectious Diseases Unit, Sheba Medical Center, Ramat Gan, Israel; ^2^ Sackler Faculty of Medicine, Tel Aviv University, Tel Aviv, Israel; ^3^ Laboratory Division, Hillel Yaffe Medical Center, Hadera, Israel; ^4^ Pediatrics Department, Bnai Zion Medical Center, Haifa, Israel; ^5^ Rappaport Faculty of Medicine, Technion Institute of Technology, Haifa, Israel; ^6^ Safra Children's Hospital, Sheba Medical Center, Ramat Gan, Israel; ^7^ Wilhelmina Children's Hospital, UMC Utrecht, Utrecht, Netherlands; ^8^ Pediatrics Department, Hillel Yaffe Medical Center, Hadera, Israel

**Keywords:** emergency medicine, pediatrics, adenovirus, host response, disease etiology

## Abstract

**Background and objectives:**

Adenovirus causes acute respiratory illness that can mimic bacterial infection, making it challenging to differentiate adenoviral infection from adenoviral-bacterial co-infection. A host-protein score (BV score) for differentiating bacterial from viral infection that combines the expression levels of TNF-related apoptosis-induced ligand, interferon gamma-induced protein-10, and C-reactive protein exhibited a negative predictive value (NPV) of 98% in prior studies. Here we evaluate BV score's diagnostic accuracy in pediatrics with adenovirus PCR detection.

**Methods:**

This is a sub-analysis of children aged 3 months to 20 years with adenovirus PCR-positive infection recruited prospectively in two previous cohort studies. Reference standard diagnosis (bacterial, viral or indeterminate) was based on expert adjudication. BV score ranges from 0 to 100 and provides three results based on predefined cutoffs: viral or other non-bacterial etiology (0 ≤ score < 35), equivocal (35 ≤ score ≤ 65), and bacterial or co-infection (65 < score ≤ 100). Experts were blinded to BV results.

**Results:**

Out of 1,779 children, 142 had an adenovirus PCR-positive nasopharyngeal swab. Median age was 1.2 years (interquartile range 0.6–1.8), 50.7% were male and 52.8% were hospitalized. 12 cases were reference standard bacterial, 115 reference standard viral and 15 were indeterminate. BV score attained sensitivity of 100.0% (no false negatives), specificity of 89.5% (95% confidence interval: 83.2–95.8), and NPV of 100.0% (92.6–100.0). Equivocal rate was 19.7%.

**Conclusions:**

BV score accurately differentiated between adenoviral and bacterial-adenoviral co-infection in this cohort of children with PCR-positive adenovirus detection. This performance supports a potential to improve appropriate antibiotic use.

## Introduction

Adenovirus is one of the major pathogens causing acute respiratory illness in children, estimated to cause 2%–5% of pediatric respiratory tract infections (RTIs) and 4%–10% of all pediatric pneumonias (1). The clinical picture of pediatric adenovirus infection is variable and includes fever, cough, tonsillitis, keratoconjunctivitis, acute otitis media, and gastroenteritis (1–5). Infections can occur sporadically throughout the year or in epidemics with a peak during winter season. Most patients affected are under the age of 5 (1, 2).

Similar to cytomegalovirus and Epstein Barr virus, adenovirus can mimic a bacterial infection, presenting with high grade and prolonged fever as well as elevated white blood cell (WBC) counts, C reactive protein (CRP) and erythrocyte sedimentation rate (ESR) (2, 3, 5, 6). Data regarding rise in procalcitonin (PCT) levels is controversial (3, 7). Specifically, the inflammatory host response triggered by adenoviruses is often characterized by elevated CRP levels substantially higher than those observed in influenza (8), which is why CRP levels are not considered a reliable indicator of bacterial co-infection for children with adenoviral infection. Generally, the bacterial-like presentation of adenoviral infection likely underlies why antibiotics are commonly mis-prescribed on admission. In a study by Chen et al. antibiotics were prescribed for more than 90% of adenovirus-positive patients during hospitalization (2). This notwithstanding, 8%–15% (9, 10) of pediatric adenoviral infections are indeed complicated by a bacterial co-infection. Since the clinical and biomarker presentation can be similar for adenoviral and adenoviral-bacterial co-infection, it is challenging to confidently diagnose adenoviral infection (6). This is the case even when multiplex polymerase chain reaction (PCR) testing is available, as adenovirus detection neither establishes active infection (11, 12) nor excludes the possibility of abacterial co-infection (10). Adenovirus can be detected in asymptomatic children (11, 12) and establish persistent/latent infection (13). Thus, the initial decision not to treat with antibiotics cannot be based on PCR results alone.

Taking together its bacterial-like presentation and the difficulty in ruling out bacterial co-infection, there is an unmet need for a diagnostic tool to assist in management of patients presenting with suspected adenoviral infection.

BV score is a host-response technology recently developed for differentiating bacterial from viral infection (14). It is based on computational integration of the circulating levels of three immune proteins: tumor necrosis factor-related apoptosis-inducing ligand (TRAIL), interferon gamma-induced protein-10 (IP-10) and CRP. Ranging from 0 to 100, the score is indicative of bacterial vs. viral infection, with defined thresholds for bacterial (or co-infection; 65 < score ≤ 100) vs. viral (or other non-bacterial; 0 ≤ score < 35) infection and equivocal scores (35 ≤ score ≤ 65). These thresholds were validated in multiple clinical studies, including a blinded study in children under 5 years old with suspected lower RTI and fever without source, where BV attained 86.7% sensitivity (95%CI: 75.8–93.1), 91.1% specificity (95%CI: 87.9–93.6), and 12.5% equivocal cases (15). Notably, when there is a bacterial-viral co-infection, BV was designed to yield a bacterial score and was demonstrated to do so across multiple virus types, including adenovirus (16). Based on multiple diagnostic accuracy studies in various clinical cohorts (14, 16–19), BV measured on a rapid, point-of-need measurement platform is CE marked and was recently FDA cleared. Its intended use is for adult and pediatric serum samples in conjunction with clinical assessments and other laboratory findings as an aid to differentiate bacterial from viral infection. The test is indicated for use in patients presenting to the emergency department (ED) or urgent care center and with samples collected at hospital admission from patients with suspected acute bacterial or viral infection, who have had symptoms for less than seven days.

Although it has a broad indication for use, BV has unique capacity to help with the challenge of discriminating between adenoviral and adenoviral-bacterial co-infection.

Here the performance of BV is examined specifically in children with adenovirus PCR detection. The study focuses on patients aged 3 months to 20 years old prospectively recruited at the ED or ward with suspected infectious disease. The diagnostic accuracy of BV was evaluated using an adjudication-based reference standard, as there is no gold standard for determining bacterial infection in the absence of positive culture findings. In addition, the potential impact of BV on antibiotic use was estimated.

## Materials and methods

### Patient population

A sub-analysis was performed on patients recruited in two prospective studies, CURIOSITY (NCT01917461) (14) and OPPORTUNITY (NCT01931254) (15). CURIOSITY recruited 1,002 patients between August 2009 and November 2013 from two hospitals in Israel. OPPORTUNITY recruited 777 patients between October 2013 and January 2015 from four hospitals in the Netherlands and two hospitals in Israel. Patients were included in the present study if they met the eligibility criteria for suspected infection of the original study, were aged 3 months to 20 years old and had adenovirus A/B/C/D/E detected by multiplex PCR in a nasal swab sample. Eligibility criteria for the original studies are given in Supplementary Methods.

### Study design

This is a sub-analysis of children enrolled in the CURIOSITY (14) and OPPORTUNITY (15) prospective observational studies. In both studies, a study-specific serum sample for BV measurements and a nasal swab sample for multiplex PCR testing for common respiratory viruses were collected from each participant. Additional tests and procedures as well as treatment were performed as needed per clinical judgement.

The entire medical record, including demographics, medical history, physical examination, laboratory, microbiology, and imaging investigation performed as part of routine care, disease course, follow-up data and study-specific serum and nasal swab results were recorded in a case report form. For detailed methods, refer to the original publications (14, 15).

### Laboratory procedures

Laboratory procedures are detailed in Supplementary Methods.

### Reference standard based on expert adjudication

The reference standard was generated based on expert panel adjudication in line with the NHS Health Technology Assessment Guidelines for Evaluation of Diagnostic Tests (20). Two or three pediatricians, each with over 7 years of experience, independently reviewed the clinical, laboratory, microbiological, radiological and follow-up data for each patient and classified each patient as: bacterial (this label includes also bacterial and viral co-infection), viral, healthy/non-infectious, or indeterminate. No guideline definitions were used by the experts for the classification of the patients. In cases where there were two experts, the discharge diagnosis in the medical record served as the third expert. Experts were blinded to one another's classifications and to BV. “Bacterial” and “viral” reference standard diagnoses were assigned when most experts gave the same classification (majority adjudication). An “indeterminate” reference standard diagnosis was assigned when either there was no majority, or most of the experts gave an indeterminate classification.

### Index test (BV score)

The index test is based on a fixed algorithm combining the expression levels of TRAIL, CRP and IP-10; the algorithm outputs a number (0–100) called BV score that is indicative of bacterial vs. viral infection (15, 17, 18, 19, 21). BV is calculated by inputting TRAIL, IP-10 and CRP measurements into the ImmunoXpert™ software (MeMed). Score cutoffs were based on manufacturer's instructions for use. The performers of BV were not provided with clinical information or reference standard data regarding the patients.

### Statistical analysis

Diagnostic performance was assessed by comparing BV to the expert adjudication reference standard, with reference standard indeterminates removed.

The statistical framework for evaluating performance required establishing that the probability of bacterial infection is an increasing function of BV. For this purpose, subjects were assigned to five pre-determined (14) score bins according to their score and within the bin according to their reference standard outcome: bacterial vs. viral/non-infectious. Objective attainment required successful pass of two statistical tests: (i) *p* < 0.05 in the Cochrane-Armitage Test for trend; and (ii) the 95% Confidence Interval (CI) of the likelihood ratio (LR) calculated for each bin should not span across the value 1 for at least 3 bins.

Sensitivity and specificity were calculated as previously (14–16). The calculations are also detailed in Supplementary Methods.

### Diagnostic error rate

The following assumptions were made to estimate the potential impact of BV on physician's practice: when BV was equivocal (35 ≤ score ≤ 65), the physician practice was unchanged, i.e., antibiotic treatment as documented in the medical record; when BV was >65 or <35 (bacterial or viral, respectively), we assumed antibiotic treatment was according to BV (i.e., full adoption by the physician). Error rate was calculated in comparison to the reference standard.

### Ethics committee approval

CURIOSITY was approved by the Hillel-Yaffe Medical Center Institutional Review Board (approval ID 0071–10-HYMC), and the Bnai-Zion Medical Center Institutional Review Board (approval ID 0084-12-BNZ). OPPORTUNITY was approved by the ethics committees in Israel and in the Netherlands.

## Results

### Patient characterization

**Figure 1:**
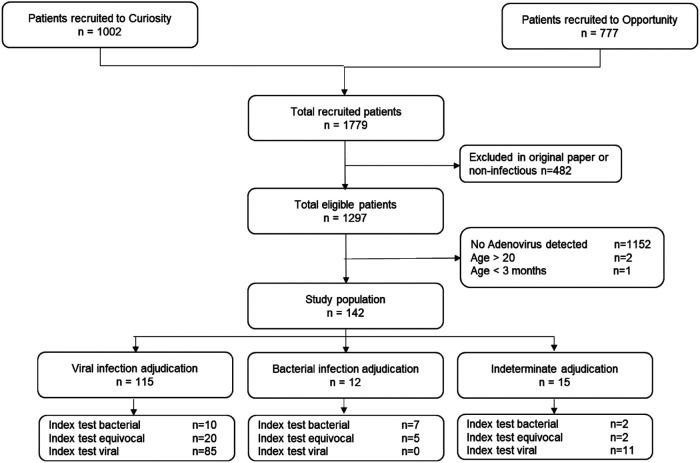
Patient enrollment flow.

Among 1,779 potentially eligible subjects from the CURIOSITY and OPPORTUNITY studies, 482 patients did not meet the original study's eligibility criteria for suspected acute infection. Among the remaining 1,297 patients, 145 had adenovirus detected by PCR (11.2%), of whom 142 satisfied age inclusion criteria; 115 cases were assigned a viral reference standard, 12 cases a bacterial reference standard (Supplementary Table 1) and 15 indeterminate (Figure 1).

Median age was 1.2 years [interquartile range (IQR) 0.6–1.8]; 50.7% were male. The children presented with relatively high-grade maximal fever, 39.4°C (38.9–39.9). The rate of hospitalized children was 52.8%, with hospital duration significantly shorter for viral vs. bacterial patients (1 vs. 2 days, respectively; *p*-value = 0.04). The viral cohort was significantly younger than the bacterial cohort (1.2, 2.6, respectively; *p* = 0.04). No other significant differences were observed between the viral and bacterial cohorts, including presenting symptoms (Table 1).

**Table 1:** Patient characterization.

	Cohort (*n* = 142)	Bacterial infection adjudication (*n* = 12)	Viral infection adjudication (*n* = 115)	Indeterminate adjudication (*n* = 15)	*P*-value*
Age (years) – median (IQR)	1.2 (0.6–1.8)	2.6 (1.2–4.0)	1.2 (0.7–1.7)	1.3 (0.6–2.0)	**0**.**038**
Gender, male – *n* (%)	72 (50.7%)	6 (50.0%)	58 (50.4%)	8 (53.3%)	1
Time from symptoms onset in days – median (IQR)	3.0 (2.0–4.0)	3.0 (2.3–3.7)	3.0 (1.5–4.5)	3.0 (2.0–4.0)	0.795
Maximal temperature in °C – median (IQR)	39.4 (38.9–39.9)	40.0 (39.6–40.4)	39.5 (39.0–40.0)	38.9 (38.2–39.6)	0.135
Hospitalized – *n* (%)	75 (52.8%)	9 (75.0%)	58 (50.4%)	8 (53.3%)	0.134
Hospitalization Duration in days – median (IQR)	1.0 (0.0–3.0)	2.0 (0.6–3.4)	1.0 (0.0–2.5)	3.0 (1.5–4.5)	**0**.**040**
Presenting symptoms – *n* (%)					
Abdominal pain	6 (4.2%)	2 (16.7%)	4 (3.5%)	0 (0.0%)	0.099
Conjunctivitis	5 (3.5%)	0 (0.0%)	5 (4.3%)	0 (0.0%)	1
Cough	77 (54.2%)	4 (33.3%)	64 (55.7%)	9 (60.0%)	0.223
Crepitations	34 (23.9%)	1 (8.3%)	28 (24.3%)	5 (33.3%)	0.294
Diarrhea	25 (17.6%)	3 (25.0%)	20 (17.4%)	2 (13.3%)	0.454
Dyspnea	23 (16.2%)	1 (8.3%)	19 (16.5%)	3 (20.0%)	0.69
Abnormal otoscopy	23 (16.2%)	2 (16.7%)	16 (13.9%)	5 (33.3%)	0.679
Pharyngitis	48 (33.8%)	5 (41.7%)	36 (31.3%)	7 (46.7%)	0.522
Sore throat	7 (4.9%)	1 (8.3%)	5 (4.3%)	1 (6.7%)	0.456
Wheezing	6 (4.2%)	1 (8.3%)	4 (3.5%)	1 (6.7%)	0.396

*P-values were calculated for bacterial cohort vs. viral cohort. Significant *P*-values (<0.05) are in bold.

### BV score performance

In the analysis cohort (*n* = 127), BV attained a sensitivity of 100.0% (95%CI: 100.0%–100.0%) specificity of 89.5% (83.2%–95.8%), and a negative predictive value (NPV) of 100.0% (92.6%–100.0%). The equivocal rate was 19.7%. To examine whether the likelihood of bacterial infection increases with score, a bin analysis was performed. The higher the score, the higher was the likelihood of a bacterial infection. More than 45% of the patients attained high confidence score results as indicated by either very low scores (0–10) or very high scores (90–100) (Table 2).

**Table 2:** Bv score performance.

Score bin	No. of patients, *n*			% of cohort			% of bin		Bacterial likelihood ratio (95% CI)
	All	B	V	All	B	V	B	V	
90 ≤ s ≤ 100	8	4	4	6.3	33.3	3.5	50.0	50.9	9.58 (2.74–33.51)
65 < s < 90	9	3	6	7.1	25.0	5.2	33.3	66.7	4.79 (1.37–16.76)
35 ≤ s ≤ 65	25	5	20	19.7	41.7	17.4	20.0	80.0	2.40 (1.10–5.22)
10 < s < 35	35	0	35	27.6	0.0	30.4	0.0	100.0	0.00 (0.00–NaN)
0 ≤ s ≤ 10	50	0	50	39.4	0.0	43.5	0.0	100.0	0.00 (0.00–NaN)
**Total**	127	12	115	100.0	100.0	100.0			

B = bacterial; V = viral; s = score.

There were no reference standard bacterial cases with a viral BV score (false negative). There were 10 cases where BV was bacterial and the reference standard was viral (false positive). Of note, the adjudication experts did not assign unanimously a viral label in 4 out of these 10 cases. Details of the false positive cases are elaborated in Supplementary Table 2.

### Comparison to routine biomarkers

**Figure 2:**
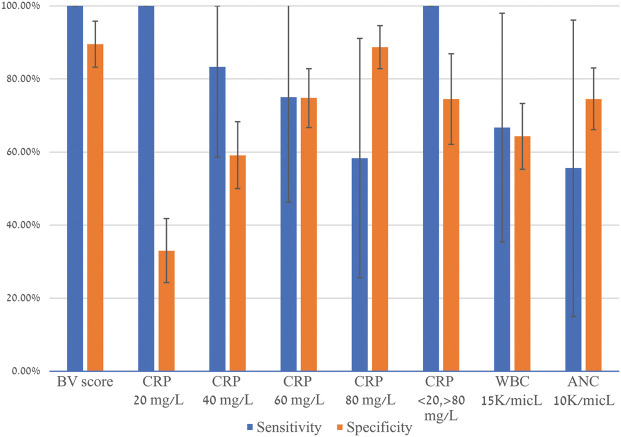
BV score comparison to routine biomarkers. Error bars indicate 95% confidence intervals. The equivocal rate was 19.7% for BV score and 54.3% for CRP 20/80. CRP, C-reactive protein; WBC, white blood cells; ANC, absolute neutrophil count.

BV outperformed routine biomarkers including CRP, WBC, and absolute neutrophil count (ANC), exhibiting higher sensitivity and/or specificity (Figure 2; Supplementary Table 3). Moreover, when CRP was applied in a rule-in/rule-out strategy, whereby below 20mg/l classifies as viral infection and above 80mg/l classifies as bacterial infection, the sensitivity was the same as BV (100.0%), but CRP's specificity was lower (74.5% vs. 89.5%, respectively) and the equivocal rate was 2.7-fold higher. Using CRP in this manner, more than 50% of the cases were not classified as bacterial or viral. Notably, out of 85 reference standard viral cases with viral BV scores (true negatives), 6 children had CRP values over 80 mg/L.

### Estimation of the impact of BV score on antibiotic use

The potential of BV to influence antibiotic use was extrapolated by comparing current antibiotic practice, as documented in the medical record, with current antibiotic practice plus BV, under the assumption that a timely, contraindicative BV score would have triggered a change in antibiotic practice, with current practice occurring in cases of equivocal scores. Applying these assumptions, it is estimated that BV could potentially reduce antibiotic prescription from 28/115 (24.3%) to 17/115 (14.8%), without significantly impacting antibiotic underuse (Supplementary Figure 1).

## Discussion

This study was performed retrospectively on children recruited in two previous prospective studies, CURIOSITY (14) and OPPORTUNITY (15), where BV performance was originally assessed on a broader population. To enable investigation of the diagnostic accuracy of BV specifically in children with confirmed adenoviral detection, children PCR-positive for adenovirus were pooled in the present sub-analysis. There were no cases classified by the adjudication-based reference standard as having a bacterial infection for which BV score was viral, i.e., no false negatives. BV outperformed routine biomarkers, including CRP, correctly identifying children with adenoviral infections (without bacterial co-infection) even when inflammatory markers were high. The diagnostic accuracy of BV was extrapolated to represent a potential reduction in antibiotic overuse of 1.6-fold, from 24.3% to 14.8%, with no significant impact on antibiotic underuse.

Adenoviral infection can present as multiple diseases (1–5). Therefore, it could be argued that a previous study that assessed BV's diagnostic accuracy in children aged 3 months to 18 years old with RTI or FWS effectively examined performance in children with suspected adenoviral infection. In the previous study, the diagnostic accuracy of BV (sensitivity 93.7% (95%CI 88.7–98.7), specificity 94.2% (92.2–96.1), PPV 73.0% (65.0–81.0), and NPV 98.9% (98.0–99.8)) was extrapolated to indicate a potential reduction in antibiotic overuse of 3.3-fold (16). It is anticipated that BV's impact on antibiotic stewardship for children presenting with signs and symptoms consistent with adenoviral infection will fall within these values. Notably, BV's impact on antibiotic prescription in the ED requires availability of the BV result within the patient's visit, which is now possible as there is a rapid sample-to-result platform. Comparability of BV results produced by the ELISA platform (employed in the studies described here) and the rapid platform has been established for serum samples (22).

Leukocytosis is considered a hallmark of bacterial infection. Herein, it was shown to perform poorly as a marker for differentiating adenoviral from adenoviral-bacterial infection, with elevated levels in 38% of the study population. Additionally, BV was demonstrated to outperform CRP, whether single cutoffs or a rule-in/rule-out strategy was applied, the latter commonly used by physicians to guide decision-making on antibiotic prescription. Further, the data indicate that application of CRP rule-in/rule-out would not only promote unwarranted antibiotic therapy, but also result in 50% of the patients yielding equivocal results whereas BV yielded 19.7% equivocal results. Of note, in the case of BV, an equivocal test result does not provide etiological information and the physician is advised to practice in line with other available patient data. Therefore, equivocal results should not impact the physician's medical decision-making and this underlies the rationale for excluding equivocal cases from calculation of sensitivity and specificity.

Notably, there are over 85 different types of human adenoviruses, classified into seven species (A–G) (23). PCR testing for adenovirus in the nasopharynx typically detects only common serotypes and in the case of lower RTIs, may not detect the virus (13). As a host-based technology that demonstrates robust performance irrespective of viral strain or infection site (14–17, 19, 21), BV addresses these limitations and can aid in management decisions for children with suspected adenoviral infection.

A strength of this study is the rigorous adjudication-based reference standard, where the experts were provided with comprehensive patient data. A potential limitation is that the experts were provided with CRP data, possibly introducing an incorporation bias in the reference standard as CRP is one of the biomarkers encompassed in the BV score. Nevertheless, various diagnostic accuracy comparisons show that BV outperformed CRP. Another limitation of this study is the small number of reference standard bacterial cases (12/127 = 9.4%), although the prevalence aligns with previous studies (9, 10). An additional limitation of the present study is that BV scores were not provided to the attending physician and therefore, the test's impact on antibiotic prescription could not be directly evaluated. Lastly, since the sub-analysis was retrospective, data regarding the physician's suspicion was not collected, precluding analysis of BV performance in children for whom the physician suspected adenoviral infection (as opposed to adenoviral-positive PCR detection). A larger prospective clinical utility study is warranted that employs the newly available rapid BV test at patient presentation. Such a study is necessary to establish BV's capacity to aid in accurately discriminating bacterial co-infection in children with suspected adenoviral infection, particularly for those who are ill-appearing, and promote appropriate management before PCR results.

In summary, BV accurately differentiated between adenoviral and bacterial-adenoviral infection in this cohort of PCR-positive adenovirus patients. Further utility studies are warranted to validate BV's potential to serve as an actionable aid when deciding on antibiotic treatment for patients with suspected adenoviral infection.

## Data Availability

Deidentified individual participant data will be made available, in addition to study protocols, the statistical analysis plan, and the informed consent form. The data will be made available upon publication to researchers who provide a methodologically sound proposal for use in achieving the goals of the approved protocol. Proposals should be submitted to Dr. Adi Klein, adik@hymc.gov.il.

## References

[1] Cherry JD Chen TK. CHAPTER 168 - ADENOVIRUSES. In: Feigin RD, Cherry JD, Demmler-Harrison GJ, Kaplan SL, editors. *Feigin and Cherry's Textbook of Pediatric Infectious Diseases (Sixth Edition)*. Philadelphia: W.B. Saunders (2009). p. 1949–72

[2] Chen S-P Huang Y-C Chiu C-H Wong K-S Huang Y-L Huang C-G Clinical features of radiologically confirmed pneumonia due to adenovirus in children. J Clin Virol. (2013) 56:7–12. 10.1016/j.jcv.2012.08.021 23021965

[3] Elenius V Peltola V Ruuskanen O Ylihärsilä M Waris M. Plasma procalcitonin levels in children with adenovirus infection. Arch Dis Child. (2012) 97:582–3. 10.1136/archdischild-2011-301308 22190671

[4] Greber UF Flatt JW. Adenovirus entry: from infection to immunity. Annu Rev Virol. (2019) 6:177–97. 10.1146/annurev-virology-092818-015550 31283442

[5] Lu M-P Ma L-Y Zheng Q Dong L-L Chen Z-M. Clinical characteristics of adenovirus associated lower respiratory tract infection in children. World J Pediatr. (2013) 9:346–9. 10.1007/s12519-013-0431-3 24235068

[6] Sun J Xiao Y Zhang M Ao T Lang S Wang J. Serum inflammatory markers in patients with adenovirus respiratory infection. Med Sci Monit. (2018) 24:3848–55. 10.12659/MSM.910692 29877315 PMC6020746

[7] Laveglia V Gorina N Cassanello P. Adenovirus infection: beware of plasma procalcitonin levels in children. Arch Dis Child. (2018) 103:622–3. 10.1136/archdischild-2017-314307 29208593

[8] Appenzeller C Ammann RA Duppenthaler A Gorgievski-Hrisoho M Aebi C. Serum C-reactive protein in children with adenovirus infection. Swiss Med Wkly. (2002) 132:345–50. 2002/25/smw-10040 12422291 10.4414/smw.2002.10040

[9] Shachor-Meyouhas Y Hadash A Kra-Oz Z Shafran E Szwarcwort-Cohen M Kassis I. Adenovirus respiratory infection among immunocompetent patients in a pediatric intensive care unit during 10-year period: co-morbidity is common. Isr Med Assoc J. (2019) 21:595–8. 31542903

[10] Song E Wang H Kajon AE Salamon D Dong S Ramilo O Diagnosis of pediatric acute adenovirus infections: is a positive PCR sufficient? Pediatr Infect Dis J. (2016) 35:827–34. 10.1097/INF.0000000000001119 26974888 PMC5292826

[11] Rhedin S Lindstrand A Rotzén-Östlund M Tolfvenstam T Öhrmalm L Rinder MR Clinical utility of PCR for common viruses in acute respiratory illness. Pediatrics. (2014) 133:e538–e45. 10.1542/peds.2013-3042 24567027

[12] Rhedin S Lindstrand A Hjelmgren A Ryd-Rinder M Öhrmalm L Tolfvenstam T Respiratory viruses associated with community-acquired pneumonia in children: matched case-control study. Thorax. (2015) 70:847–53. 10.1136/thoraxjnl-2015-206933 26077969

[13] Biserni GB Scarpini S Dondi A Biagi C Pierantoni L Masetti R Potential diagnostic and prognostic biomarkers for adenovirus respiratory infection in children and young adults. Viruses. (2021) 13:1885. 10.3390/v13091885 34578465 PMC8472906

[14] Oved K Cohen A Boico O Navon R Friedman T Etshtein L A novel host-proteome signature for distinguishing between acute bacterial and viral infections. Schildgen O, editor. PLoS One. (2015) 10:e0120012. 10.1371/journal.pone.0120012 25785720 PMC4364938

[15] van Houten CB de Groot JAH Klein A Srugo I Chistyakov I de Waal W A host-protein based assay to differentiate between bacterial and viral infections in preschool children (OPPORTUNITY): a double-blind, multicentre, validation study. Lancet Infect Dis. (2017) 17:431–40. 10.1016/S1473-3099(16)30519-9 28012942

[16] Papan C Argentiero A Porwoll M Hakim U Farinelli E Testa I A host signature based on TRAIL, IP-10, and CRP for reducing antibiotic overuse in children by differentiating bacterial from viral infections: a prospective, multicentre cohort study. Clin Microbiol Infect. (2022) 28:723–30. 10.1016/j.cmi.2021.10.019 34768022

[17] Stein M Lipman-Arens S Oved K Cohen A Bamberger E Navon R A novel host-protein assay outperforms routine parameters for distinguishing between bacterial and viral lower respiratory tract infections. Diagn. Microbiol. Infect. Dis. (2018) 90:206–13. 10.1016/j.diagmicrobio.2017.11.011 29273482

[18] Ashkenazi-Hoffnung L Oved K Navon R Friedman T Boico O Paz M A host-protein signature is superior to other biomarkers for differentiating between bacterial and viral disease in patients with respiratory infection and fever without source: a prospective observational study. Eur J Clin Microbiol Infect Dis. (2018) 37:1361–71. 10.1007/s10096-018-3261-3 29700762 PMC6015097

[19] Srugo I Klein A Stein M Golan-Shany O Kerem N Chistyakov I Validation of a novel assay to distinguish bacterial and viral infections. Pediatrics. (2017) 140:e20163453. 10.1542/peds.2016-3453 28904072

[20] Rutjes AWS Reitsma JB Coomarasamy A Khan KS Bossuyt PMM. Evaluation of diagnostic tests when there is no gold standard. A review of methods. Health Technol Assess. (2007) 11:iii, ix -51. 10.3310/hta11500 18021577

[21] Eden E Srugo I Gottlieb T Navon R Boico O Cohen A Diagnostic accuracy of a TRAIL, IP-10 and CRP combination for discriminating bacterial and viral etiologies at the emergency department. J Infect. (2016) 73:177–80. 10.1016/j.jinf.2016.05.002 27255416

[22] Hainrichson M Avni N Eden E Feigin P Gelman A Halabi S A point-of-need platform for rapid measurement of a host-protein score that differentiates bacterial from viral infection: Analytical evaluation. Clin Biochem. (2022) S0009-9120(22)00115-1. 10.1016/j.clinbiochem.2022.04.012 35487256

[23] Dhingra A Hage E Ganzenmueller T Böttcher S Hofmann J Hamprecht K Molecular evolution of human adenovirus (HAdV) Species C. Sci Rep. (2019) 9:1039. 10.1038/s41598-018-37249-4 30705303 PMC6355881

