# Supplementary material for: BV score differentiates viral from bacterial-viral co-infection in adenovirus PCR positive children
Source: Front Pediatr. 2022 Nov 1;10:990750. doi: 10.3389/fped.2022.990750 (PMC9664934; doi:10.3389/fped.2022.990750)
Supplement: Supplementary file 1 [file Datasheet1.pdf]

Supplementary Material

CONTENTS

1 SUPPLEMENTARY METHODS 2

1.1 Eligibility criteria employed in the original studies 2

1.2 Laboratory procedures 2

1.3 Statistical analysis 2

2 SUPPLEMENTARY TABLES 4

2.1 Supplementary Table 1. Reference standard bacterial cases (n=12) 4

2.2 Supplementary Table 2. Cases where the BV score was bacterial and the reference standard was viral (false positives, n=10) 8

2.3 Supplementary Table 3. BV score comparison to routine biomarkers 11

3 SUPPLEMENTARY FIGURES 12

3.1 Supplementary Figure 1. Potential impact of BV score on antibiotic overuse and underuse. 12

4 REFERENCES 13

1 SUPPLEMENTARY METHODS

1.1 Eligibility criteria employed in the original studies

CURIOSITY [1]

Inclusion criteria: clinical suspicion of an acute infectious disease, peak fever >37.5°C since onset of symptoms, and duration of symptoms ≤12 days.

Exclusion criteria: evidence of acute infection in the two weeks preceding enrollment; congenital immune deficiency; treatment with immunosuppressive or immunomodulatory therapy; active malignancy; and human immunodeficiency virus (HIV), or hepatitis B/C virus infection.

OPPORTUNITY [2]

Inclusion criteria: patients aged between 2 and 60 months presenting with fever (peak temperature ≥38.0°C measured by axillary, rectal, or ear thermometer) and symptoms of lower respiratory tract infection or fever without source existing for a maximum of 6 days.

Exclusion criteria: previous episode of fever in the past 3 weeks; psychomotor retardation; moderate to severe metabolic disorder; primary or secondary immunodeficiency; HIV, infection by hepatitis B or hepatitis C viruses; and active malignancies.

1.2 Laboratory procedures

In the CURIOSITY study, nasal swabs were stored at 4°C for up to 72h before transport to a central laboratory, where two multiplex PCR analyses were conducted to detect common respiratory viral (Seeplex RV15) and bacterial (Seeplex PB6) pathogens. Venous blood specimens were stored at 4°C for up to 5h before fractionation into serum and plasma and storage at −80°C. Host-protein biomarkers were measured as follows: CRP using either Cobas-6000, Cobas-Integra-400/800, or Modular-Analytics- P800 (Roche); TRAIL and IP-10 using ImmunoXpert™ (MeMed).
In the OPPORTUNITY study, nasal swabs were collected (universal transport medium, Copan, Brescia, Italy), frozen within 2h and stored at -80°C before transport to the MeMed laboratory, where multiplex PCR testing was performed for 15 common respiratory viruses (Seeplex RV15, Seegene, Seoul, South Korea). Similarly, venous blood specimens were fractionated into serum and plasma within 2h of collection and stored at −80°C before transport to the MeMed laboratory; TRAIL and IP-10 measurements were performed using ImmunoXpert™ (MeMed) and CRP was measured using Cobas e501 or Cobas-6000.

1.3 Statistical analysis

Sensitivity was defined as the number of patients that received a bacterial reference standard and a bacterial BV score (score>65), divided by the number of patients that received a bacterial reference standard. Specificity was symmetrically defined as the number of patients that received a viral reference standard and a viral BV score (score<35), divided by the number of patients that received a viral reference standard. The percentage of cases assigned an equivocal score (35≤score≤65) was reported. Fisher's exact test was used for comparing proportions.

Positive predictive value (PPV) was defined as the number of patients that received a bacterial reference standard and a bacterial index test score (score > 65), divided by the number of patients that received a bacterial index test score. Negative predictive value (NPV) was symmetrically defined as the number of patients that received a viral reference standard and a viral index test score (score < 35), divided by the number of patients that received a viral index test score.

2 SUPPLEMENTARY TABLES

2.1 Supplementary Table 1. Reference standard bacterial cases (n=12)

<table>
<tr><td rowspan="4">Patient 627</td><td>BV</td><td>Experts</td><td>Sex</td><td>Age (y)</td><td>Discharge diagnosis</td><td>Max temp</td><td>Hospitalization Duration</td><td>Abx</td></tr>
<tr><td>100</td><td>Bacterial</td><td>F</td><td>20</td><td>Gastroenteritis</td><td>40</td><td>2</td><td>Ciproxin</td></tr>
<tr><td colspan="3">Microbiology</td><td>CXR</td><td>LAB</td><td>CRP</td><td colspan="2">Clinical details</td></tr>
<tr><td colspan="3">Positive Adenovirus. Negative stool cultures.</td><td>Normal</td><td>WBC 12.5K, ANC 11K</td><td>346.9</td><td colspan="2">Fever and vomiting 3 days before admission. Normal physical examination.</td></tr>
<tr><td rowspan="4">Patient 3887</td><td>BV</td><td>Experts</td><td>Sex</td><td>Age (y)</td><td>Discharge diagnosis</td><td>Max temp</td><td>Hospitalization Duration</td><td>Abx</td></tr>
<tr><td>98</td><td>Bacterial</td><td>F</td><td>0.7</td><td>Pyelonephritis</td><td>40.5</td><td>6</td><td>Augmentin, Gentamycin, Ceftriaxon, Co-trimoxazol</td></tr>
<tr><td colspan="2">Microbiology</td><td>CXR</td><td>LAB</td><td>CRP</td><td colspan="3">Clinical details</td></tr>
<tr><td colspan="2">Positive Adenovirus and Bocavirus. Positive E.coli urine culture. Negative blood culture.</td><td>NA</td><td>WBC 32K, ANC NA</td><td>122.2</td><td colspan="3">Fever and vomiting 1 day before admission. In physical examination - ill-appearing, without any other significant sign. In US, unilateral enlarged pyelum, indicative of pyelonephritis.</td></tr>
<tr><td rowspan="4">Patient 591</td><td>BV</td><td>Experts</td><td>Sex</td><td>Age (y)</td><td>Discharge diagnosis</td><td>Max temp</td><td>Hospitalization Duration</td><td>Abx</td></tr>
<tr><td>97</td><td>Bacterial</td><td>F</td><td>16</td><td>Acute Tonsillitis</td><td>39.2</td><td>2</td><td>During hospitalization with Penicillin G, Discharged with Moxypen</td></tr>
<tr><td colspan="2">Microbiology</td><td>CXR</td><td>LAB</td><td>CRP</td><td colspan="3">Clinical details</td></tr>
<tr><td colspan="2">Positive Adenovirus. Negative blood culture. Positive Group G strep nasopharyngeal culture. Positive Mycoplasma pneumoniae (MP) IgM, EBV negative IgM, CMV negative IgM positive IgG, Q-fever negative IgM.</td><td>NA</td><td>WBC 10K, ANC 7K</td><td>135.9</td><td colspan="3">Fever and sore throat 3 weeks before admission. In physical examination enlarged tonsils and enlarged cervical lymph nodes.</td></tr>
<tr><td>Patient 479</td><td>BV</td><td>Experts</td><td>Sex</td><td>Age (y)</td><td>Discharge diagnosis</td><td>Max temp</td><td>Hospitalization Duration</td><td>Abx</td></tr>
</table>

	97	Bacterial	M	3.5	Pneumonia	39	2	During hospitalization with Cefuroxime, Discharged with Moxypen
	**Microbiology**		**CXR**	**LAB**	**CRP**	**Clinical details**		
	Positive Adenovirus and Parainfluenza virus 4. Positive Streptococcus pneumoniae (SP) and Haemophilus influenza (HI) PCR. Negative Mycoplasma pneumoniae (MP) IgM.		LLL consolidation	WBC 33K, ANC 30K	85.8	Fever, cough and vomiting from day of admission. In physical examination decreased breath sounds at the base left lung.		
**Patient 3530**	**BV**	**Experts**	**Sex**	**Age (y)**	**Discharge diagnosis**	**Max temp**	**Hospitalization Duration**	**Abx**
	89	Bacterial	M	2	Pneumonia	40	4	Treated with Azenil prior to admission, treates with Ampicillin during hospitalization, Discharged with Moxypen
	**Microbiology**		**CXR**	**LAB**	**CRP**	**Clinical details**		
	Positive Adenovirus. Negative blood culture.		RUL Consolidation	WBC 27K, ANC 18K	105.0	Fever and cough 5 day before admission. In physical examination decreased breath sounds at the right lung.		
**Patient 483**	**BV**	**Experts**	**Sex**	**Age (y)**	**Discharge diagnosis**	**Max temp**	**Hospitalization Duration**	**Abx**
	81	Bacterial	M	2.2	Acute Tonsillitis	40.7	0	Discharged with Moxypen
	**Microbiology**		**CXR**	**LAB**	**CRP**	**Clinical details**		
	Positive Adenovirus. Positive Streptococcus pneumoniae (SP) and Haemophilus influenza (HI) PCR. Positive Mycoplasma pneumoniae (MP) IgM, EBV CMV negative IgM igG. Negative blood culture.		Normal	WBC 19K, ANC 13K	20.6	Fever 1 day before admission. In physical examination, bilateral TM erythema and enlarged tonsils with exudate.		
**Patient 3349**	**BV**	**Experts**	**Sex**	**Age (y)**	**Discharge diagnosis**	**Max temp**	**Hospitalization Duration**	**Abx**

	75	Bacterial	F	3.2	Cellulitis	NA	3	Treates with Moxypen prior to admission, treates with Cefamezin during hospitalization, Discharged with Ceforal
	**Microbiology**		**CXR**	**LAB**	**CRP**	**Clinical details**		
	Positive Adenovirus. Negative blood and urine cultures.		Normal	WBC 21K, ANC 13K	78.3	Fever and cellulitis 2 days before admission. In physical examination angular erythema above the left ankle and erythematous tonsils.		
**Patient 702**	**BV**	**Experts**	**Sex**	**Age (y)**	**Discharge diagnosis**	**Max temp**	**Hospitalization Duration**	**Abx**
	62	Bacterial	F	5	Kawasaki; Gastroenteritis	40.3	5	NA
	**Microbiology**		**CXR**	**LAB**	**CRP**	**Clinical details**		
	Positive campylobacter stool culture, positive Adenovirus, negative urine, blood and nasopharyngeal culture, EBV CMV negative IgM igG, positive Haemophilus influenza (HI) PCR		NA	WBC 8K, ANC 5K	44.9	Fever, vomiting and diarrhea 5 days before admission, rash from day of admission. Physical examination: diffuse maculopapular rash, non purulent bilateral conjunctivitis, strawberry tongue and cracked lips. enlarged unilateral lymph node, systolic heart murmur. Treated with IVIG and aspirin for Kawasaki disease.		
**Patient 3796**	**BV**	**Experts**	**Sex**	**Age (y)**	**Discharge diagnosis**	**Max temp**	**Hospitalization Duration**	**Abx**
	58	Bacterial	F	3	AOM	39.01	2	Treates with Augmentin prior to admission, Augmentin treatment was continued.
	**Microbiology**		**CXR**	**LAB**	**CRP**	**Clinical details**		
	Positive Adenovirus		NA	WBC 13K, ANC 11	173.5	Fever, apathy and otodynia 3 days before admission. In physical examination - ill-appearing, unilateral ear bulging, enlarged tonsils		
**Patient 351**	**BV**	**Experts**	**Sex**	**Age (y)**	**Discharge diagnosis**	**Max temp**	**Hospitalization Duration**	**Abx**
	56	Bacterial	M	1.5	Pneumonia	40	NA	Discharged with Moxypen

<table>
<tr><td rowspan="2"></td><td colspan="2">Microbiology</td><td>CXR</td><td>LAB</td><td>CRP</td><td colspan="3">Clinical details</td></tr>
<tr><td colspan="2">Positive Adenovirus, positive GAS nasopharyngeal culture, Negative blood culture. Normal urine stick, positive Streptococcus pneumoniae (SP) and Haemophilus influenza (HI) PCR.</td><td>LLL consolidation</td><td>WBC 15K, ANC 7K</td><td>112.0</td><td colspan="3">Fever 3 days before admission, Known backgroung of minimal hydronephrosis. In physical examination enlarged tonsils with exudate.</td></tr>
<tr><td rowspan="4">Patient 963</td><td>BV</td><td>Experts</td><td>Sex</td><td>Age (y)</td><td>Discharge diagnosis</td><td>Max temp</td><td>Hospitalization Duration</td><td>Abx</td></tr>
<tr><td>53</td><td>Bacterial</td><td>M</td><td>0.5</td><td>Gastroenteritis</td><td>40</td><td>NA</td><td>Discharged with Azenil</td></tr>
<tr><td colspan="2">Microbiology</td><td>CXR</td><td>LAB</td><td>CRP</td><td colspan="3">Clinical details</td></tr>
<tr><td colspan="2">Positive campylobacter stool culture, positive Adenovirus, positive Streptococcus pneumoniae (SP) PCR</td><td>NA</td><td>WBC 15K, ANC 9K</td><td>74.5</td><td colspan="3">Fever and diarrhea 5 days before admission, on day of admission bloody stool. Normal physical examination</td></tr>
<tr><td rowspan="4">Patient 3745</td><td>BV</td><td>Experts</td><td>Sex</td><td>Age (y)</td><td>Discharge diagnosis</td><td>Max temp</td><td>Hospitalization Duration</td><td>Abx</td></tr>
<tr><td>49</td><td>Bacterial</td><td>M</td><td>0.9</td><td>Bronchiolitis</td><td>38.5</td><td>13</td><td>Augmentin</td></tr>
<tr><td colspan="2">Microbiology</td><td>CXR</td><td>LAB</td><td>CRP</td><td colspan="3">Clinical details</td></tr>
<tr><td colspan="2">Positive RSV, Adenovirus, HMPV, Bocavirus PCR, Positive Adenovirus Ag in stool, negative Noro and Rota virus stool Ag</td><td>Bilateral pneumonia and atelectasis</td><td>WBC 24K (max 28K), ANC 20K</td><td>26.4</td><td colspan="3">Fever, dyspnea, cough and rhinoreaha 2 days before admission. Background of prematurity and IRDS. In physical examination - ill-appearing, saturation 85%, rales, crepitation, wheezing, prolonged expiration and accessory muscle use. Was hospitalized in PICU and intubated, lowest saturation 48%.. Complaints and lab resolved without intervention. Follow up - still hospitalized in PICU</td></tr>
</table>

2.2 Supplementary Table 2. Cases where the BV score was bacterial and the reference standard was viral (false positives, n=10)

<table>
<tr><td rowspan="4">Patient 3539</td><td>BV</td><td>Experts</td><td>Sex</td><td>Age (y)</td><td>Discharge diagnosis</td><td>Max temp</td><td>Hospitalization Duration</td><td>Abx</td></tr>
<tr><td>99</td><td>Viral</td><td>M</td><td>1</td><td>Upper respiratory tract infection</td><td>39.2</td><td>2</td><td>Not given</td></tr>
<tr><td colspan="3">Microbiology</td><td>CXR</td><td>LAB</td><td colspan="3">Clinical details</td></tr>
<tr><td colspan="3">Positive Adenovirus, Enterovirus and Rhinovirus
Negative blood culture</td><td>LLL consolidation (official result), RML consolidation in hospitalization summary, in revision- no finding.</td><td>WBC 7.63K, ANC 0.6K</td><td colspan="3">Fever, cough and rhinorrhea a day before admission. In physical examination, moaning, bronchial breath sounds.</td></tr>
<tr><td rowspan="4">Patient 1255</td><td>BV</td><td>Experts</td><td>Sex</td><td>Age (y)</td><td>Discharge diagnosis</td><td>Max temp</td><td>Hospitalization Duration</td><td>Abx</td></tr>
<tr><td>96</td><td>Viral</td><td>M</td><td>2.5</td><td>Acute tonsillitis</td><td>39.8</td><td>4</td><td>Not given</td></tr>
<tr><td colspan="3">Microbiology</td><td>CXR</td><td>LAB</td><td colspan="3">Clinical details</td></tr>
<tr><td colspan="3">Positive Adenovirus
Negative blood culture</td><td>Normal</td><td>WBC 13K, ANC 10K</td><td colspan="3">Fever and vomiting 4 days before admission. Background of prematurity. Erythema in pharynx in physical examination. Complaints and lab resolved without intervention.</td></tr>
<tr><td rowspan="4">Patient 1337</td><td>BV</td><td>Experts</td><td>Sex</td><td>Age (y)</td><td>Discharge diagnosis</td><td>Max temp</td><td>Hospitalization Duration</td><td>Abx</td></tr>
<tr><td>93</td><td>Viral</td><td>M</td><td>1.5</td><td>Gastroenteritis</td><td>39.5</td><td>3</td><td>Ceftriaxone IV</td></tr>
<tr><td colspan="3">Microbiology</td><td>CXR</td><td>LAB</td><td colspan="3">Clinical details</td></tr>
<tr><td colspan="3">Positive Adenovirus, Coronavirus OC43 and Rhinovirus
Negative blood and stool culture.</td><td>Not done</td><td>WBC 26K, ANC 13K</td><td colspan="3">Fever, vomiting and diarrhea a day before admission. Physical examination normal except for lymphadenopathy. Improvement after treatment.</td></tr>
<tr><td rowspan="3">Patient 3125</td><td>BV</td><td>Experts</td><td>Sex</td><td>Age (y)</td><td>Discharge diagnosis</td><td>Max temp</td><td>Hospitalization Duration</td><td>Abx</td></tr>
<tr><td>92</td><td>Viral</td><td>F</td><td>0.45</td><td>Bronchiolitis with secondary bacterial infection</td><td>38.5</td><td>4</td><td>Treated with Moxypen, discharged with Moxypen</td></tr>
<tr><td colspan="3">Microbiology</td><td>CXR</td><td>LAB</td><td colspan="3">Clinical details</td></tr>
</table>

<table>
<tr><td></td><td colspan="3">Positive Adenovirus and parainfluenza, negative Influenza and RSV
Negative blood culture</td><td>Normal</td><td>WBC 13K, ANC 6</td><td colspan="3">Fever and dyspnea 4 days before admission. In physical examination dyspnea and tachypnea, diffuse rales, prolonged expiration, decreased breathing sounds.</td></tr>
<tr><td rowspan="4">Patient 1360</td><td>BV</td><td>Experts</td><td>Sex</td><td>Age (y)</td><td>Discharge diagnosis</td><td>Max temp</td><td>Hospitalization Duration</td><td>Abx</td></tr>
<tr><td>89</td><td>Viral</td><td>M</td><td>4.5</td><td>Acute tonsillitis</td><td>39.1</td><td>4</td><td>Ampicillin IV was started and stopped after appearance of rash.</td></tr>
<tr><td colspan="3">Microbiology</td><td>CXR</td><td>LAB</td><td colspan="3">Clinical details</td></tr>
<tr><td colspan="3">Positive Adenovirus
Negative nasopharyngeal, blood and urine culture, EBV, CMV IgG positive, IgM negative</td><td>Normal</td><td>WBC 5K, ANC 3K</td><td colspan="3">Fever, cough, sore throat and abdominal pain a week before admission. In physical examination enlarged tonsils with exudate, lymphadenopathy, splenomegaly. Abx was started and stopped due to rash which was suspected as viral infection.</td></tr>
<tr><td rowspan="4">Patient 3955</td><td>BV</td><td>Experts</td><td>Sex</td><td>Age (y)</td><td>Discharge diagnosis</td><td>Max temp</td><td>Hospitalization Duration</td><td>Abx</td></tr>
<tr><td>84</td><td>Viral</td><td>F</td><td>2</td><td>Upper respiratory tract infection</td><td>38.7</td><td>2</td><td>Not given</td></tr>
<tr><td colspan="3">Microbiology</td><td>CXR</td><td>LAB</td><td colspan="3">Clinical details</td></tr>
<tr><td colspan="3">Positive Adenovirus, Enterovirus and Rhinovirus</td><td>Not done</td><td>WBC unknown, ANC unknown</td><td colspan="3">Fever, cough and dyspnea a day before admission. In physical examination accessory muscle use, rales and prolonged expiration.</td></tr>
<tr><td rowspan="4">Patient 563</td><td>BV</td><td>Experts</td><td>Sex</td><td>Age (y)</td><td>Discharge diagnosis</td><td>Max temp</td><td>Hospitalization Duration</td><td>Abx</td></tr>
<tr><td>81</td><td>Viral</td><td>M</td><td>1.5</td><td>Acute tonsillitis</td><td>41</td><td>Unknown</td><td>Not given</td></tr>
<tr><td colspan="3">Microbiology</td><td>CXR</td><td>LAB</td><td colspan="3">Clinical details</td></tr>
<tr><td colspan="3">Positive Adenovirus</td><td>Normal</td><td>WBC 17K, ANC 9K</td><td colspan="3">Fever, cough, rhinorrhea and decreased appetite 3 days before admission. HRAD background. In physical examination ill apearing, erythema in pharynx and upper respiratory rales. Was hospitalized for observation, improved without treatment.</td></tr>
<tr><td rowspan="2">Patient 3056</td><td>BV</td><td>Experts</td><td>Sex</td><td>Age (y)</td><td>Discharge diagnosis</td><td>Max temp</td><td>Hospitalization Duration</td><td>Abx</td></tr>
<tr><td>78</td><td>Viral</td><td>M</td><td>1.75</td><td>Acute tonsillitis</td><td>40</td><td>0</td><td>Not given</td></tr>
</table>

	**Microbiology**			**CXR**	**LAB**	**Clinical details**		
	Positive Adenovirus and Parainfluenza<br>Negative nasopharyngeal and blood culture culture			Normal	WBC 27K, ANC 8.4K	Fever, dyspnea, dry cough and decreased appetite 3 days before admission. Known background of recurrent tonsilitis and AOM. In physical examination enlarged tonsils with exudate and upper respiratory rales. No findings of abscess in ENT examination.		
**Patient 3477**	**BV**	**Experts**	**Sex**	**Age (y)**	**Discharge diagnosis**	**Max temp**	**Hospitalization Duration**	**Abx**
	75	Viral	F	2	Unspecified viral infection	38.5	2	Not given
	**Microbiology**			**CXR**	**LAB**	**Clinical details**		
	Positive Adenovirus and Rhinovirus			Not done	WBC 6.3K	Fever and rhinorrhea 3 days before admission. Vomiting from day of admission. Parent with acute tonsillitis treated with abx. Suspected AOM in physical examination, in second examination no finding.		
**Patient 672**	**BV**	**Experts**	**Sex**	**Age (y)**	**Discharge diagnosis**	**Max temp**	**Hospitalization Duration**	**Abx**
	72	Viral	F	1	Gastroenteritis	38.5	3	Not given
	**Microbiology**			**CXR**	**LAB**	**Clinical details**		
	Positive Adenovirus, Bocavirus, Rhinovirus, Streptococcus pneumoniae and Haemophilus influenza. Positive Norovirus in stool PCR, positive Clostridium difficile Toxin B, Negative stool and blood culture			Not done	WBC 10K, ANC 5K	Fever, vomiting and diarrhea, 4 days before admission contacted ED, was diagnosed as viral GE, treated with fluids and discharged home. Returned to the ED because of no improvement. known background of congenital hypothyroidism. In physical examination: conjunctivitis. Admitted and treated with fluids, improvement after treatment.		

2.3 Supplementary Table 3. BV score comparison to routine biomarkers

	n = 127	Sensitivity % (95% CI)	Specificity % (95% CI)	PPV % (95% CI)	NPV % (95% CI)	Equivocal rate %
**Score**	**< 35 or > 65**	100.0 (100.0-100.0)	89.5% (83.2-95.8)	41.2 (15.1-67.3)	100.0 (92.6-100.0)	**19.7**
**CRP**	**20**	100.0 (100.0-100.0)	33.0 (24.3-41.8)	13.5 (6.2-20.7)	100.0 (52.6-100.0)	
	**40**	83.3 (58.6-100.0)	59.1 (50.0-68.3)	17.5 (7.4-27.7)	97.1 (78.9-100.0)	
	**60**	75.0 (46.3-100.0)	74.8 (66.7-82.8)	23.7 (9.5-37.8)	96.6 (84.9-100.0)	
	**80**	58.3 (25.6-91.1)	88.7 (82.8-94.6)	35.0 (12.1-57.9)	95.3 (88.9-100.0)	
	**< 20 or > 80**	100.0 (100.0-100.0)	74.5 (62.1-86.9)	35.0 (12.1-57.9)	100.0 (80.5-100.0)	**54.3**
**WBC**	**15,000**	66.7 (35.4-98.0)	64.3 (55.3-73.3)	16.7 (5.7-27.6)	94.7 (78.9-100.0)	
**ANC**	**10,000**	55.6 (15.0-96.1)	74.5 (66.1-83.0)	15.6 (2.3-28.9)	95.2 (83.2-100.)	

CRP, C-reactive protein; WBC, white blood cells; ANC, absolute neutrophil count; PPV, positive predictive value; NPV, negative predictive value.

3 SUPPLEMENTARY FIGURES

3.1 Supplementary Figure 1. Potential impact of BV score on antibiotic overuse and underuse.

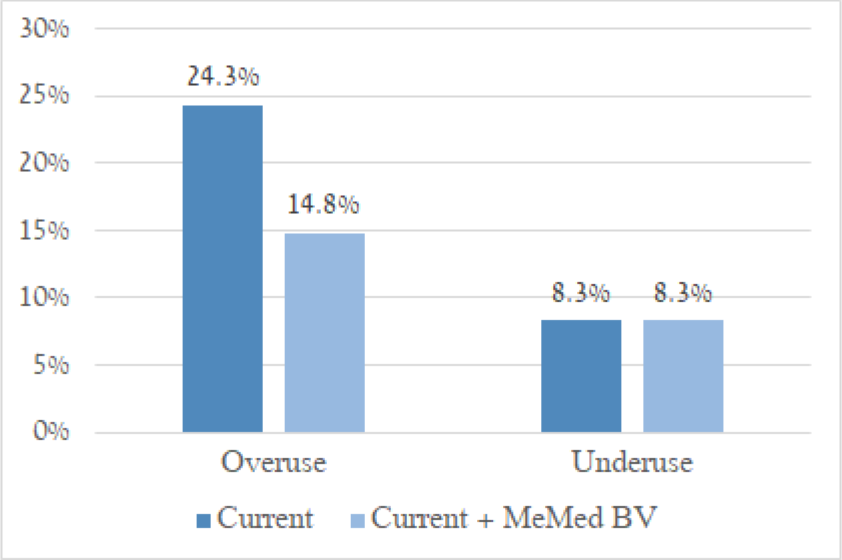

The calculations and assumptions are explained in Methods. Data is shown for the analysis cohort; n=127.

4 REFERENCES

1 Oved K, Cohen A, Boico O, *et al.* A Novel Host-Proteome Signature for Distinguishing between Acute Bacterial and Viral Infections. *PLoS ONE* 2015;**10**:e0120012. doi:10.1371/journal.pone.0120012

2 van Houten CB, de Groot JAH, Klein A, *et al.* A host-protein based assay to differentiate between bacterial and viral infections in preschool children (OPPORTUNITY): a double-blind, multicentre, validation study. *Lancet Infect Dis* 2017;**17**:431–40. doi:10.1016/S1473-3099(16)30519-9
